# Heterogeneous Nuclear Ribonucleoprotein K Interacts with Abi-1 at Postsynaptic Sites and Modulates Dendritic Spine Morphology

**DOI:** 10.1371/journal.pone.0027045

**Published:** 2011-11-15

**Authors:** Christian Proepper, Konrad Steinestel, Michael J. Schmeisser, Jutta Heinrich, Julie Steinestel, Juergen Bockmann, Stefan Liebau, Tobias M. Boeckers

**Affiliations:** 1 Institute for Anatomy and Cell Biology, Ulm University, Ulm, Germany; 2 Department of Pathology, BWK Hospital Ulm, Ulm, Germany; Federal University of Rio de Janeiro, Brazil

## Abstract

**Background:**

Abelson-interacting protein 1 (Abi-1) plays an important role for dendritic branching and synapse formation in the central nervous system. It is localized at the postsynaptic density (PSD) and rapidly translocates to the nucleus upon synaptic stimulation. At PSDs Abi-1 is in a complex with several other proteins including WASP/WAVE or cortactin thereby regulating the actin cytoskeleton via the Arp 2/3 complex.

**Principal Findings:**

We identified heterogeneous nuclear ribonucleoprotein K (hnRNPK), a 65 kDa ssDNA/RNA-binding-protein that is involved in multiple intracellular signaling cascades, as a binding partner of Abi-1 at postsynaptic sites. The interaction with the Abi-1 SH3 domain is mediated by the hnRNPK-interaction (KI) domain. We further show that during brain development, hnRNPK expression becomes more and more restricted to granule cells of the cerebellum and hippocampal neurons where it localizes in the cell nucleus as well as in the spine/dendritic compartment. The downregulation of hnRNPK in cultured hippocampal neurons by RNAi results in an enlarged dendritic tree and a significant increase in filopodia formation. This is accompanied by a decrease in the number of mature synapses. Both effects therefore mimic the neuronal morphology after downregulation of Abi-1 mRNA in neurons.

**Conclusions:**

Our findings demonstrate a novel interplay between hnRNPK and Abi-1 in the nucleus and at synaptic sites and show obvious similarities regarding both protein knockdown phenotypes. This indicates that hnRNPK and Abi-1 act synergistic in a multiprotein complex that regulates the crucial balance between filopodia formation and synaptic maturation in neurons.

## Introduction

Synaptogenesis describes a multistep procedure leading to polarized cell-cell contacts that transmit information from one neuron to the other [1], [2]. Synapses are mainly formed during early brain development but they are also lost, newly established and/or altered in morphology during life time. These plastic changes, closely related to environmental factors as well as to synaptic activity are thought to be the morphological bases for memory formation in the CNS [3], [4]. Up to now, however, it is not completely clear how these local structural changes are induced and which synaptic molecules, signaling pathways and mechanisms are involved [5]. The local rearrangement of actin filaments to a branched actin meshwork that is accomplished by actin-nucleating factors (ANFs) and/or small GTPases is certainly an essential step for the maturation of synaptic spines from primitive filopodia to mushroom-shaped synapses with a fully established postsynaptic density (PSD) [6], [7], [8]. The Abelson-interacting protein 1 (Abi-1) is a 68-kDa protein [9] that has been shown to interact with the scaffolding postsynaptic density (PSD-) protein ProSAP2/Shank3 via a C-terminal *src*-homology (SH3) domain as well as with the eponymous Abelson tyrosine kinase [10]. Abi-1 is localized in neuritic growth cones and in later developmental state in dendritic spines and PSDs where it plays an important role in regulating cytoskeletal reorganization and synaptic maturation [11]. It is already known that the *wave-binding* (WAB) *domain* of Abi-1 is responsible for the specific interaction with WAVE1, a WASP/WAVE family initiation factor of actin polymerization through activation of small GTPases [12], [13]. Furthermore, it has been demonstrated that Abi-1 - together with Nap1, PIR121 and HSPC300 - is part of a stable multiprotein complex that is capable of binding to and thus activating WAVE family proteins [14]. Taken together, these findings support an important role of Abi-1 in the regulation of actin dynamics as one part of a multiprotein complex interacting with actin-polymerizing factors such as the WAVE protein family. Upon synaptic activation, Abi-1 translocates into the nucleus where it might act as a transcription factor in complex with Myc/Max proteins [10]. These different tasks are mediated by functionally different protein domains of the Abi-1 protein, such as the WAVE-binding domain (WAB), a DNA-binding homeobox homology region (HHR) or the aforementioned C-terminal *src*-homology (SH3) domain. We performed a yeast two-hybrid-screen with a human fetal brain cDNA-library using a full-size Abi-1 cDNA as the bait to identify novel interaction partners of Abi-1. We were especially interested in proteins that might be involved in Abi-1 transport or function in different cell compartments and/or Abi-1 effects on synaptogenesis and actin reorganization. The yeast two hybrid screen revealed several independent clones coding for the *heterogeneous nuclear ribonucleoprotein K* (hnRNPK), a ribonucleoprotein with a molecular weight of 65 kDa. hnRNPK has been isolated from multiple tissues, such as fibroblasts, neurons and epithelial cells, and is believed to act mostly as an essential part of RNP complexes that are important for pre- mRNA processing and transport. hnRNPK is able to bind single or double stranded nucleic acids, especially in CU/CT-rich regions via its three *K homology* (KH-) domains [15], [16], [17]. Furthermore, it contains a *nuclear localization signal* (NLS) and a *nuclear shuttling domain* (KNS) [18]. Several proteins are already known to bind to the hnRNPK (KI-) domain [15], [19], thus modulating mRNA binding affinity. Interestingly, hnRNPK has also been identified as a direct interaction partner of N-WASP via KI/WH1-domain-interaction [20]. In this study, it could be shown that hnRNPK suppresses filopodia formation in spreading cells, while it stimulates N-WASP-mediated actin polymerization in an *in vitro* assay. Based on these data, a regulatory role for hnRNPK in N-WASP-mediated actin polymerization is proposed. The hnRNPK ribonucleoprotein complex is a target of several intracellular signaling cascades [21], [22] and phosphorylation of the protein modulates mRNA binding, thus affecting translation directly or via mRNA stabilization [23], [24]. Various examples of these post-transcriptional regulation processes through hnRNPK have been elucidated [25], [26], [27], [28]. In 2002, Ostareck-Lederer et al. described the regulation of DICE-dependent translation of 15-lipoxygenase (15-LOX) through c-src phosphorylation of hnRNPK in erythroid precursor cells [29]. Protein Kinase C (PKC) phosphorylates hnRNPK on amino acid S302 in renal proximal tubular epithelial cells, conveying hnRNPK binding to vascular endothelial growth factor (vEGF) mRNA [30]. This leads to enhanced translation of the mRNA in response to elevated extracellular angiotensin II levels. Furthermore, it has been shown that hnRNPK modulates the expression of neurofilament mRNAs during development of the cerebral cortex [31], [32].

Our data show that Abi-1 and hnRNPK colocalize in primary CNS neurons at synaptic sites. This protein interaction is mediated via an Abi-1-SH3/hnRNPK-KI-domain interplay. Interestingly, the downregulation of hnRNPK results in extensive filopodia formation and an enlargement of the dendritic tree, a morphologic phenotype that is similar to the one created by Abi-1 knockdown. Moreover, mature synapse formation is reduced so that our data support the growing knowledge for a central role of hnRNPK in actin dynamics as a prerequisite for dendritic spine formation and synaptic maturation.

## Materials and Methods

### Ethics Statement

All animal experiments were performed in compliance with the guidelines for the welfare of experimental animals issued by the Federal Government of Germany, the National Institutes of Health and the Max Planck Society. The experiments in this study were approved by the review board of the Land Baden Württemberg, Permit Number Nr. O.103.

### Antibodies

The following primary antibodies were used in this study: anti-GFP (Clontech, San Diego, CA), anti-Myc (Roche, Mannheim, Germany), anti-hnRNPK (Abnova, Heidelberg, Germany), anti-Abi-1 (MBL, Nagoya, Japan), anti-Bassoon (Stressgen/Enzo Life Sciences, Lörrach, Germany), anti-beta-Actin (Sigma-Aldrich, Steinheim, Germany), anti-IgG (Miltenyi Biotech, Bergisch-Gladbach, Germany), anti-ProSAP2/Shank3 [10].

### Yeast two-hybrid screen

A yeast two-hybrid screen was performed using the Y187 und AH109 yeast strains harboring the reporter genes HIS3 and β-galactosidase (β-gal) under the control of an upstream GAL1 activating sequence. The YTH screen was carried out with the full coding sequence of Abi-1 as bait that was fused in frame to the GAL4 DNA binding domain in the pAS2-1 vector (Clontech) and transformed to screen against a human fetal brain cDNA-library cloned into the pACT2 vector (Clontech). The two hybrid screen was carried out according to the manufacturer's protocol. A total of 2×10^6^ cotransformants were screened, yeast colonies that grew in medium lacking histidine were picked up, and their ß-galactosidase activity was assayed by X-gal filter-lift assays. The cDNAs of transformants which turned blue within 4 hours in the initial test and after re-transformation were isolated and sequenced. Nine independent hnRNPK clones could be identified.

### Cell culture

Cell culture experiments of rat hippocampal primary neurons (embryonic day 18–21; E18–21) were performed as described previously [33]. In brief: After preparation, the hippocampal neurons were seeded on poly-L-lysine (0,1 mg/ml; Sigma-Aldrich, Steinheim, Germany) coated coverslips at a density of 4×10^4^ cells/well (transfection experiments) or 2×10^4^ cells/well (immunocytochemical staining). Cells were grown in Neurobasal medium, complemented with B27 supplement, 0,5 mM L-glutamine and 100 U/ml penicillin/streptomycin (all Invitrogen, Karlsruhe, Germany) and maintained at 37°C in 5% CO_2_. Hippocampal cells were transfected on the days indicated, using Lipofectamine 2000 according to the manufacturer's recommendation (Invitrogen). Cos7 and NIH3T3 cells (obtained from DSMZ, Braunschweig, Germany) were maintained in Dulbecco's modified Eagle's medium (DMEM) with high glucose (Invitrogen), supplemented with 10% (v/v) fetal calf serum and 2 mM L-glutamine without antibiotics. Cells were grown on commercially available chamber-slides (Nunc, Wiesbaden, Germany) treated with poly-L-lysine (0,1 mg/ml; Sigma-Aldrich). Transfection experiments were performed using the transfection-agent Fugene (Roche) according to the manufacturer's recommendations. 22 hours after transfection, cells were fixed with 4% paraformaldehyde and processed for indirect immunofluorescence.

### Expression vectors and transfection experiments

To verify the interaction between Abi-1 and hnRNPK in Cos7 cells, subregions of Abi-1 (AbiSH3 and AbiΔSH3) and hnRNPK (K1-K3) were subcloned into the appropriate GFP and Myc expression vectors (pEGFP and pCMV-Myc, Clontech) using PCR strategies. Afterwards, different combinations of hnRNPK and Abi-1-expression constructs were cotransfected into Cos7 cells. In these transfection-experiments the following plasmids were used: hnRNPK-K1-GFP (aa1–149), expressing the nuclear localization signal (NLS) and the K homology 1 (KH1) regions fused to GFP; hnRNPK-K2-GFP (aa150–337), expressing the KH2 and K interaction (KI) domains; hnRNPK-K3-GFP (aa337–464), expressing the nuclear shuttling domain (KNS) and KH3 domains, hnRNPK-full-GFP and -Myc (aa1–464; full-size hnRNPK fused to GFP and Myc), Abi-1-GFP and Abi-1-Myc (aa1–476; full-size Abi-1), Abi-1-SH3-Myc (aa417–476), Abi-1ΔSH3-Myc (aa1–416; Abi-1 without the C-terminal SH3-domain). Additionally, we generated an RNAi resistant hnRNPK full size plasmid using the eGFP-C1 vector by mutating the binding site of the hnRNPK RNAi2 construct as follows: by exchanging AGT CTG with TCA TTG (bp 460–465) leading to no changes in the amino acid sequence. The site directed mutagenesis kit was used (Stratagene, La Jolla, USA). All used PCR fragments and clones were analyzed by DNA-sequencing.

### Subfractionation protocol

Tissue fractionation was performed essentially as described by Carlin et al. [34] with some modifications [35], [36], [37]. In brief, tissue from 21 day old rats was homogenized in homogenization buffer (320 mM sucrose, 5 mM HEPES, pH 7.4) containing protease inhibitor mixture (Roche) to obtain the crude brain lysate (Homogenate). Cell debris and nuclei were removed by centrifugation at 1000× g. The supernatant was spun for 20 min at 12.0000× g resulting in supernatant S2 (soluble fraction, mainly cytosolic proteins) and pellet P2 (crude synaptosomal fraction, membrane-associated proteins). P2 was further fractionated by centrifugation in a sucrose step gradient (0.85, 1.0 and 1.2 M) for 2 h at 200.000× g resulting in the myelin-enriched fraction (located on top of the gradient), the light membrane fraction (0.85/1.0 interphase) and the purified synaptosomal fraction (1.0/1.2 interphase). For isolation of synaptic junctional proteins, the purified synaptosomal fraction was diluted with 5 volumes of 1 mM Tris pH 8.1 and stirred on ice for 30 min. After centrifugation for 30 min at 33.000× g, the pellet P3 was resuspended in 5 mM Tris pH 8.1 and once again fractionated by centrifugation in a sucrose gradient for another 2 h at 200.000× g. The 1.0/1.2 M interphase (synaptic junctions) was suspended in 320 mM sucrose, 0.5% Triton X-100, 5 mM Tris pH 8.1, stirred on ice for 15 min and centrifuged for 30 min at 33.000× g resulting in the first PSD pellet (PSD I, one triton-extracted PSD fraction). For further purification, this pellet was resuspended in the same aforementioned 0.5% Triton X-100 containing buffer, stirred on ice for 15 min and finally centrifuged for another 30 min at 33.000× g resulting in the twice triton-extracted PSD fraction (PSD II).

### Immunoprecipitation of proteins and Western blot analysis

For immunoprecipitation experiments, 2 µg of primary antibody was preincubated for 1 h at 4°C with 50 µl of magnetic microbeads (μMACS Micro Beads; Miltenyi Biotech). Protein lysates were incubated with antibody-coupled microbeads for 1 h on ice. As a negative control, we incubated lysate and microbeads without any antibody or used unspecific anti-IgG antibodies. Alternatively, anti GFP/anti-Myc microbeads were incubated with lysate of transfected Cos7 cells. As control, untransfected Cos7 cells were tested. Probes were loaded on μMACS-microcolums and washed 10 times with washing-buffer; proteins were eluted with DTT-probe buffer before loading on a SDS-gel. Homogenates from different organs or brain regions and/or immunoprecipitated proteins were separated by SDS-page, blotted on PVDF membranes and incubated with the appropriate primary antibody. Immunoreactivity was visualized by HRP-conjugated secondary antibodies (DAKO A/S, Denmark) and Super-Signal West Pico chemoluminescence (Pierce, Rockford, USA).

### Immunocytochemistry and immunohistochemistry

Immunofluorescence was performed according to [38], [39]. In brief, primary cultures were fixed with ice-cold 4% paraformaldehyde (PFA)/1,5% sucrose/PBS for 20 min at 4°C and processed for immunocytochemistry. After washing three times with 1×PBS for 5 min at RT, the cells were permeabilized for 3 min on ice in a buffer containing 0,1% Triton X-100/0,1% Na-Citrate/PBS and washed again 3 times with 1×PBS. Blocking was performed with 10% FCS/PBS for 1 h at RT followed by incubation with the primary antibody o/n at RT. After a further washing-step, the cells were incubated with the secondary antibody coupled to Alexa488 (green) or Alexa568 (red) (Invitrogen) for 90 min at RT, washed first with 1×PBS and then with aqua bidest for 5 min and mounted in Mowiol (with or without DAPI for staining of the nucleus). Neuronal morphology was analyzed using an upright Axioskop microscope equipped with a Zeiss CCD camera (16 bits; 1280×1024 pixels per image), and further processed using Axiovision software (Zeiss) and Adobe Photoshop software (Adobe Systems, San Jose, CA). Immunohistochemical staining of rat brain was performed using 7 µm microtome sections, which were fixed by immersion in Bouin's fluid for 48 h, dehydrated, and embedded in paraplast. hnRNPK was detected either with a mouse anti hnRNPK monoclonal or polyclonal antibody diluted 1∶400 using the avidin-biotin-peroxidase complex (ABC) technique. Briefly, the ABC method included incubation of cryostat, deparaffinized sections with primary antibodies in PBS containing 0.1% Triton X-100 for 24 h at RT, followed by incubation for 2 h at RT with the secondary antibody, biotin-labeled goat anti-mouse IgG (Jackson ImmunoResearch; West Grove, USA). The sections were then incubated for 2 h with a preformed complex of biotin-peroxidase-streptavidin (Jackson ImmunoResearch), and peroxidase activity was revealed with 0.02% diaminobenzidine hydrochloride (DAB) as chromogen.

### In situ hybridization

In situ hybridization was performed essentially as described previously [33]. Transcripts encoding hnRNPK were detected with a S^35^ labeled cDNA antisense oligonucleotide directed against the 3′ end of the mRNA (GGG CTC CAT GTA TCT ATT GCA GAG TCC CAA GT;bp1061); purchased from MWG-Biotech (Ebersberg, Germany).

### Small interference RNA (RNAi) experiments

Knockdown of Abi-1 or hnRNPK was achieved by RNAi according to published methods using the pSuper vector (OligoEngine, Seattle, WA). For this plasmid-based RNA inhibition of Abi-1, the following complementary oligonucleotides were annealed and inserted into the *Hin*d III/*Bgl* II sites of the pSUPER vector: 5′-GAT CCC CAG GCT ACA GAC AAG AGG AAT TCA AGA GAT TCC TCT TGT CTG TAG CCT TTT TTG GAA A-3′ and 5′- AGC TTT TCC AAA AAA GGC TAC AGA CAA GAG GAA TCT CTT GAA TTC CTC TTG TCT GTA GCC TGG G-3′ (rat Abi-1; Acc.Nr NM_024397.2). For hnRNPK, an RNAi construct targeting the 3′ untranslated Region (hnRNPK RNAi1) was designed with the following sequence: 5′-GAT CCC CGT GCA CCT CTT GGA TAT AGT TCA AGA GAC TAT ATC CAA GAG GTG CAC TTT TTG GAA A-3′ and 5′-AGC TTT TCC AAA AAG TGC ACC TCT TGG ATA TAG TCT CTT GAA CTA TAT CCA AGA GGT GCA CGG G-3′. (rat hnRNPK; Acc.Nr NM_057141). A second RNAi construct (hnRNPK RNAi2) was generated targeting the cDNA of hnRNPK with the following sequence: 5′-GAT CCC CAG TCT GGC CGG AGG AAT TAT TCA AGA GAT AAT TCC TCC GGC CAG ACT TTT TTG GAA A-′3 and 5′-AGC TTT TCC AAA AAA GTC TGG CCG GAG GAA TTA TCT CTT GAA TAA TTC CTC CGG CCA GAC TGG G-′3. NIH3T3 cells as well as hippocampal neurons were transfected with the pSuper RNAi-hnRNPK, or pSuper RNAi-Abi-1 constructs. Control cells were obtained by transfecting the empty pSuper vector.

## Results

### Abi-1 interacts with hnRNPK via its SH3 domain

**Figure 1:**
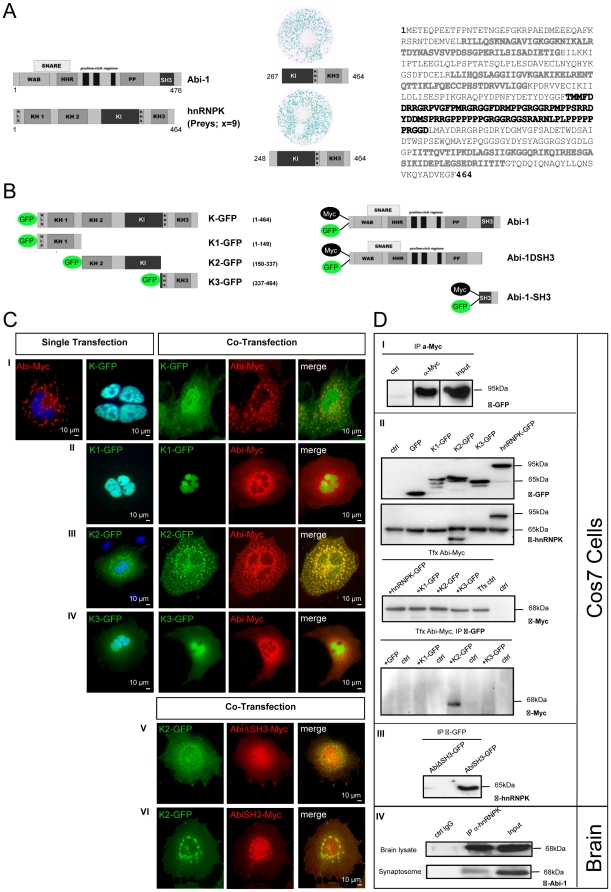
hnRNPK interacts with Abi-1 via its KH2 domain. (A) Yeast two-hybrid screen. The full length Abi-1 cDNA was cloned as bait to screen a human fetal brain cDNA-library for putative interaction partners. 9 independent partial C-terminal hnRNPK clones were identified and retested for interaction by a yeast two-hybrid assay. Results are shown for the longest (aa248–464) and shortest (aa267–464) prey clone. hnRNPK is a 464 aa long protein that codes for several specific domains: N-terminal NLS, *nuclear localization signal*, KH1-KH3, *K homology domains 1–3 (light grey)*; KI, *K interaction domain (black)*; KNS, *K nuclear shuttling signal*. Abi-1 (476 aa) codes for the following domains: WAB, *WAVE binding domain*; SNARE, HHR, *homeobox homology region*; PP, *proline rich domain*; SH3 *src homology 3 domain*. (B) Schematic illustration of the Abi-1 and hnRNPK clones (and abbreviations) that have been used for further experiments. (C) The hnRNPK KH2 domain colocalizes with Abi-1.Several partial GFP- or Myc-tagged hnRNPK and Abi-1 clones were coexpressed in Cos7 cells to identify the interacting subdomains of the two proteins. In single transfection experiments, hnRNPK full length protein predominantly localizes to the nucleus, whereas Abi-1 shows a typical cytoplasmic staining pattern. When coexpressed, both proteins are localized in identical dotted structures (I). In contrast, the K1-recombinant protein alone is restricted to the nucleus and does not colocalize with Abi-1 after cotransfection (II). K2 fusion protein readily colocalizes with full-size Abi-1 in the cytoplasm (III). As shown for K1, K3 also shows no colocalization with Abi-1 (IV). The cotransfection of hnRNPK K2 with Abi-1 missing the SH3 domain (AbiΔSH3) results in no colocalization (V), the expression of Abi-1 SH3 domain alone (AbiSH3), however, gives rise to a perfect overlay in the perinuclear region (VI). (D) Coimmunoprecipitation experiments with overexpressed and endogenous Abi-1 and hnRNPK proteins. Plasmids encoding full-size hnRNPK-GFP and Abi-1-Myc were cotransfected in Cos7 cells and Abi-1-Myc was immobilized using anti-Myc microbeads loaded on a column. Protein-complexes then were eluted, separated by SDS-Page and hnRNPK-GFP (size 95 kDA) was detected by immunoblot using a specific anti-GFP antibody (I). As controls, beads loaded with lysate only (ctrl) and the input lysate were used. (II) Cos7 cells were transfected with partial hnRNPK-coding constructs K1-GFP (KH1 domain), K2-GFP (KH2 and KI domain), K3-GFP (KH3 domain) and K-full-GFP (full length) as GFP-fusion proteins. The correct expression of the hnRNPK constructs was controlled by using an anti-GFP antibody and a commercial anti-hnRNPK antibody that could detect the GFP fusion protein as well as the endogenous hnRNPK in the lysate (95 kDA and 65 kDA). Moreover, the commercial antibody detects the K2 construct. The correct expression and antibody specificity of the Abi-1-Myc construct was tested by cotransfection with truncated hnRNPK constructs and subsequent immunoblotting with an anti-Myc antibody. Afterwards, precipitation was performed with GFP-tagged microbeads after cotransfection of Abi-1-Myc and hnRNPK constructs. The precipitates were subjected to immunostaining with an anti-Myc antibody. The Abi-1-Myc protein could only be detected within hnRNPK-K2-GFP precipitate but not within K1-GFP and K3-GFP precipitate or within the GFP-only and/or negative controls. (III) *Vice versa* experiments were done by coimmunoprecipitations using lysates of Cos7 cells cotransfected with a combination of full length hnRNPK-Myc (K-Myc) and AbiΔSH3-GFP or AbiSH3-GFP, respectively. The immunoprecipitation was performed using antibodies directed against GFP and immunoblot-detection was performed using anti-hnRNPK antibodies showing that expression of the Abi-1 SH3 domain is a prerequisite for protein binding. (IV) The hnRNPK antibody was used to precipitate the protein complex from brain lysate as well as from the synaptosomal fraction. In the Western blot, an antibody against Abi-1 could readily detect its antigen in the precipitate. As positive control, brain lysate or synaptosomal material was used (Input lane: 4% of the total lysate used for immunoprecipitation), a negative control was performed with unspecific IgG (ctrl IgG). Scale bars are as indicated.

A yeast two-hybrid (YTH) screen has been performed to identify novel interaction partners of Abi-1. Among other cDNAs, we could isolate 9 independent clones coding for the heterogeneous nuclear ribonucleoprotein K (hnRNPK) (Fig. 1A, the amino acid sequence). After retesting this candidate protein in yeast, we performed several transfection experiments with hnRNPK- and Abi-1 -GFP and -Myc expression constructs in Cos7 cells (Fig. 1B,C). The single-transfected recombinant Abi-1-Myc protein reveals a punctate cytoplasmic staining pattern, while hnRNPK-GFP expression seems to be restricted to the nucleus. However, a cytoplasmic colocalization of Abi-1-Myc and hnRNPK-GFP can be recognized when both full-length constructs are cotransfected into Cos7 cells (Fig. 1C-I). The recombinant hnRNPK-K1 protein (K1-GFP: NLS and KH1 domain) localizes exclusively to the nucleus and shows neither redistribution nor colocalization when coexpressed with full length Abi-1-Myc (Fig. 1C-II), while hnRNPK-K2 (K2-GFP: KH2 and KI domain) reveals a perfect colocalization with recombinant Abi-1-Myc (Fig. 1C-III).

Furthermore, there is no colocalization of hnRNPK-K3 (K3-GFP: KH3 domain and KNS) and Abi-1 (Fig. 1C-IV). The hnRNPK-K2-GFP fusion protein does not colocalize with an Abi-1-Myc protein lacking the C-terminal SH3 domain (Abi-1ΔSH3-Myc), whereas a perfect colocalization with the Abi-1 SH3 domain alone can be detected, indicating the direct interaction of hnRNPK's K2 domain with the Abi-1 SH3 domain (Fig. 1C-V and VI). In further transfection experiments in Cos7 cells with recombinant hnRNPK-GFP/Abi-1-Myc, we could precipitate hnRNPK-GFP via Abi-1-Myc from Cos7 cell lysate after co-transfection (Fig. 1D-I). After testing the expression and immunoreactivity of our constructs, we conducted coimmunoprecipitation experiments in which a precipitation of the hnRNPK-K2 domain but not K1 or K3 via immobilized Abi-1-Myc could be detected (Fig. 1D-II). These results could be confirmed by a precipitation assay which showed that “full-length” hnRNPK coimmuno-precipitates with the SH3 domain of Abi-1 while there is no complex formation with Abi-1ΔSH3 (Fig. 1D-III). Finally, Abi-1 could be coimmuno-precipitated from both total brain lysate and the synaptosomal fraction with specific hnRNPK antibodies linked to magnetic beads (Fig. 1D-IV).

### hnRNPK mRNA and protein reveal developmental stage dependent expression pattern in the brain

**Figure 2:**
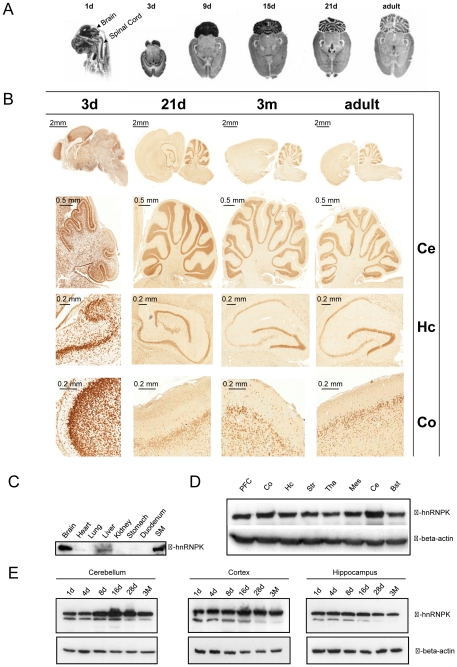
Expression pattern of hnRNPK in the developing CNS. (A) In situ hybridization of hnRNPK mRNA during rat brain development. At embryonic time points (1d), the mRNA of hnRNPK can easily be detected in all areas of the developing brain including the spinal cord. At later stages of maturation (horizontal sections, 3d-adult), the expression levels decrease and become more and more restricted to the cortex (Co), the hippocampal formation (Hc) and the granular layer of the cerebellum (Ce). (B) Immunohistochemical detection of hnRNPK in rat brain sagittal sections. At early time points of brain development (3d), a predominant nuclear labeling of hnRNPK can be detected in nearly all neurons. Again, cortex, hippocampus and cerebellum are most strongly labeled. At later time points, differences in spatial expression become even more prominent and intense staining is especially seen in granule cells of the cerebellum (Ce) and the dentate gyrus as well as in the CA1-4 regions of the hippocampus. In the cortex (Co), the staining intensity diminishes at later stages and only some scattered neurons in deeper cortical layers remain positive for hnRNPK. (C) Analysis of hnRNPK expression in different tissues and organs. hnRNPK is readily detectable in brain, liver and skeletal muscle (SM) while heart as well as lung, kidney, stomach and duodenum are almost devoid of detectable hnRNPK. (D) The analysis of hnRNPK expression in different brain areas (9d). A comparable expression profile of the protein is seen in the prefrontal cortex (PFC), the parietal cortex (Co), hippocampus (Hc), striatum (Str), thalamus (Tha), mesencephalon (Mes), and in the brain stem (Bst) while cerebellum (Ce) shows highest expression levels. Loading control: Actin. (E) Western blot analysis of time dependent hnRNPK expression in selected brain regions. hnRNPK detection in cerebellum, cortex and hippocampus at different stages of maturation shows that in all regions investigated, the strong signal at 8 to 28d becomes slightly weaker at 3 M. Loading control: Actin. Scale bars are as indicated.

*In situ* hybridization of hnRNPK mRNA detects the highest expression in cerebellum, the hippocampus and cortical regions of the developing rat nervous system, with a cerebellar expression peak around postnatal day 9 (9d). Here, the mRNA is mainly expressed in the granule cell layer of the cerebellum. There is also a strong mRNA expression in the hippocampal region throughout all investigated developmental stages. In general, the mRNA levels in brain seem to be reduced at older stages and are more and more restricted within the above mentioned brain areas (Fig. 2A). This observation is supported by immunostainings with hnRNPK antibodies. The pyramidal cells within the CA region as well as granule cells of the dentate gyrus of the hippocampal formation (Hc) show a peak of expression of mostly nuclear localized hnRNPK around day 3, and stay stable during later stages of maturation. In the cerebellum (Ce) hnRNPK is localized predominantly in the granular cell layer. During the first developmental days from day 3 to day 21, hnRNPK expression in neurons of the cortex (Co) decreases and is only observed in deeper cortical layers (Fig. 2B). The analysis of different rat tissues revealed that the highest levels of hnRNPK protein expression can be found in brain and skeletal muscle. Interestingly, besides liver tissue, most other organs were devoid of or contained very low levels of hnRNPK protein (Fig. 2C). Western Blot analysis of distinct brain areas at day 9 (9d) reveals that at this developmental stage, all subcompartments of the brain contain hnRNPK protein while cerebellum exhibits highest levels of expression thus reflecting the *in situ* results (Fig. 2D). A closer analysis of protein expression by Western Blot in various brain regions during postnatal brain maturation supports the immunohistochemical data by showing a reduction of hnRNPK immunoreactivity especially of the lower molecular weight isoform of hnRNPK running at approx. 60 kDa (Fig. 2E).

### Neuronal hnRNPK localizes to the nucleus and to postsynaptic sites

**Figure 3:**
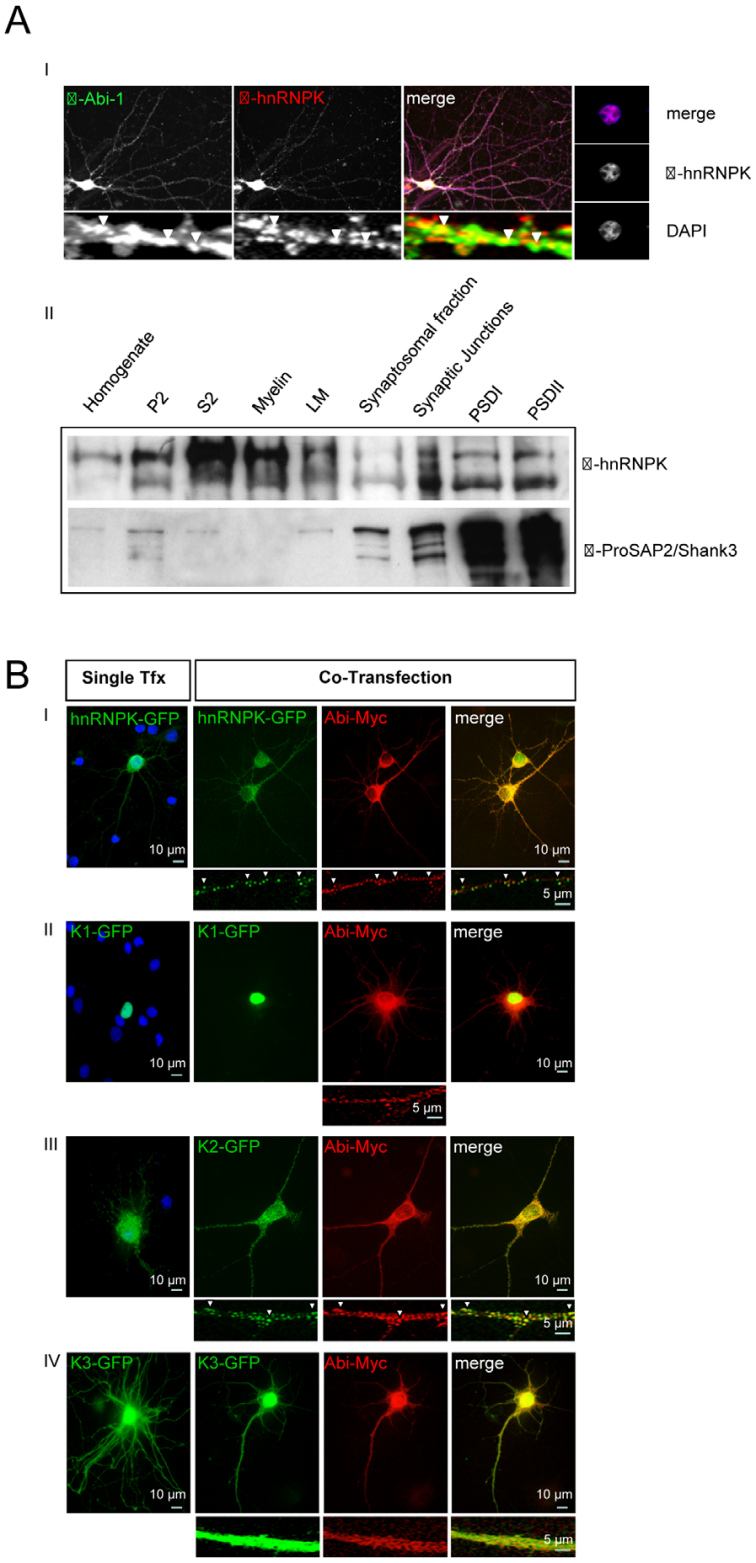
hnRNPK shows nuclear and postsynaptic localization that depends on Abi-1 interaction. (A) In hippocampal neurons cultured for 12 days (DIV12), hnRNPK is distributed predominantly in the nucleus (magnified right panel) but can also be stained in the dendritic compartment. Here it colocalizes with Abi-1 (arrowheads) that is highly enriched within the postsynaptic density (PSD) (I). Western Blot analysis of neuronal subfractions shows the appearance of the hnRNPK positive band not only in the synaptosomal and synaptic junctional subfractions, but also within the PSD fractions supporting its localization within the postsynaptic subcompartment. As purification control, the neuronal subfractions were also stained for ProSAP2/Shank3, a protein that is highly enriched within the postsynaptic density (II). Homogenate = crude brain lysate P2 = crude synaptosomal fraction, S2 = soluble fraction, Myelin = myelin-enriched fraction, LM = light membrane fraction, PSDI = one triton-extracted, PSDII = twice triton-extracted postsynaptic density fraction(s). 10 µg of protein was loaded for each lane. (B) When hnRNPK-GFP and Abi-1-Myc fusion proteins are coexpressed in hippocampal neurons (DIV21), a clear colocalization at synaptic sites can be detected (I). The same result can be seen after cotransfection and expression of Abi-1-Myc and K2-GFP, which encodes the KI interaction domain, initially shown as the binding region for Abi-1 (III). Here, the colocalization can be detected in perinuclear clusters and at Abi-1-positive postsynaptic sites. Therefore, this interaction site seems to be sufficient for postsynaptic localization of the hnRNPK protein. In contrast, the recombinant K1-GFP or K3-GFP reveal no colocalization with cotransfected Abi-1-Myc after transfection in hippocampal neurons (II,IV). Scale bars are as indicated.

In immunostainings of hippocampal neurons with antibodies directed against Abi-1 and hnRNPK, we detected hnRNPK to be predominantly found in the nucleus as shown by costaining with DAPI, but with a clear overlay of the immunofluorescence for both proteins in the PSD of the dendritic cell compartment (Fig. 3A-I). This was underlined by Western Blot analysis of brain subcellular fractions showing that hnRNPK protein is indeed a component of the synaptic PSD fraction (Fig. 3A-II). To analyze the mechanisms which target hnRNPK to postsynaptic sites, we carried out a series of transfection experiments with the assortment of clones that had already been characterized in the Cos7 cell experiments (Fig. 1B,C). In hippocampal neurons, cultured for 21 days, recombinant hnRNPK-GFP and Abi-1-Myc showed an identical localization at synaptic sites (Fig. 3B-I). When the interaction domain of hnRNPK (K2-GFP) is cotransfected together with Abi-1, this hnRNPK-GFP residual protein is also perfectly targeted to Abi-1 positive synapses (Fig. 3B-III). However, a nuclear and/or cytoplasmic distribution and no colocalization could be found in cotransfection experiments of Abi-1 with hnRNPK-K1-GFP or K3-GFP constructs (Fig. 3B-II, 3B-IV). These results support the finding that an Abi-1 interaction with the hnRNPK KI-domain (encoded by hnRNPK-construct K2) is responsible for the synaptic localization of hnRNPK in neurons.

### Effects of hnRNPK knockdown on neuronal morphology

**Figure 4:**
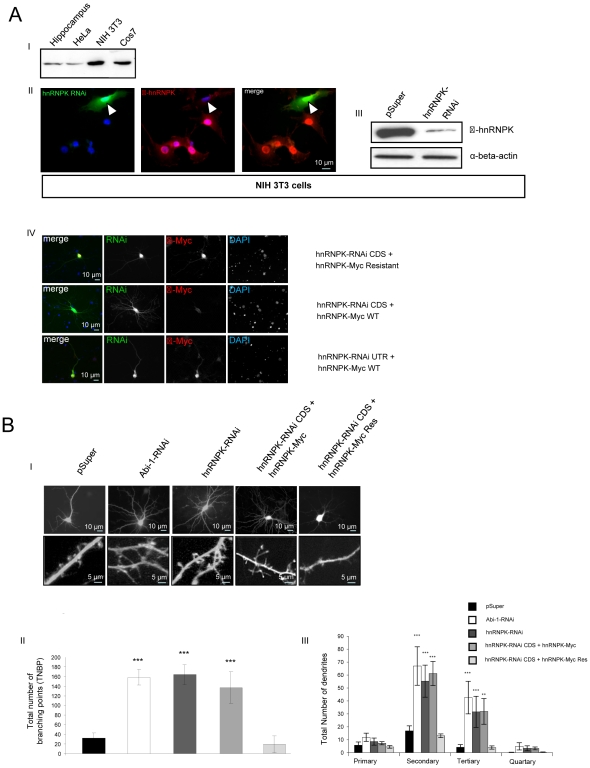
Downregulation of hnRNPK mimics the “Abi-1 depletion phenotype” in neurons. (A) hnRNPK-RNAi knockdown in NIH3T3 cells with a construct targeting the 3′UTR-region of hnRNPK. (I) hnRNPK is endogenously expressed in NIH3T3, HeLA and Cos7 cells and is detectable in cultured hippocampal neurons (DIV21). (II) After transfection of NIH3T3 cells with an hnRNPK-RNAi construct for 3 days, the cells were fixed and stained with an antibody against hnRNPK. Only untransfected cells in close proximity to the RNAi-transfected cell in the upper right are immunopositive for hnRNPK, with a predominant distribution of the protein in the nucleus. (III) After transfection of NIH3T3 cells with an hnRNPK-RNAi construct for 3 days, protein expression of hnRNPK is markedly suppressed as confirmed by Western blotting compared to vector control transfected cells. (IV) Double transfections using two different hnRNPK-RNAi constructs, one targeting the 3′UTR of the hnRNPK sequence and one targeting the coding sequence of hnRNPK together with an hnRNPK-Myc-construct which is resistant against RNAi due to 4 nucleotide exchanges in the RNA leading to an unaltered amino acid sequence. The staining against the Myc-tag shows a decreased protein level solely when using the non-resistant construct together with the RNAi targeting the CDS (coding sequence). (B) Neuronal transfection of Abi-1-RNAi and hnRNPK-RNAi constructs. (I) In contrast to the control vector (pSuper), transfection of RNAi constructs resulted in an obvious change of neuronal morphology. The downregulation of hnRNPK as well as Abi-1 is leading to an extended and extremely branched dendritic tree. (II) The number of branching points within the dendritic compartment is significantly upregulated in both RNAi groups compared to the control transfection. (III) The analysis of the dendritic tree shows a significant shift of dendrites towards small, filopodia-like tertiary dendrites. Scale bars are as indicated.

Using freshly prepared cell-lysates from different cell lines, we investigated in a non-quantitative manner that hnRNPK is endogenously expressed in rat hippocampal neurons (21 days *in vitro*, DIV21), HeLa cells, NIH3T3 mouse fibroblasts and Cos7 cells (Fig. 4A-I). Based on these data, we first tested the knockdown properties of the hnRNPK-RNAi constructs in cell lines, which endogenously express hnRNPK. After transfection and immunocytochemical staining, RNAi-transfected NIH3T3 cells were nearly depleted of hnRNPK, confirmed by Western blotting (Fig. 4A-II and -III). The specific knock-down and the functionality of the RNAi-resistant hnRNPK clone was verified by immunocytochemistry using different combinations of double transfection. We found hnRNPK protein from the RNAi resistant construct is expressed despite cotransfection of specific RNAi-constructs targeting either the 3′UTR or the coding sequence of hnRNPK. In contrast, when the non-resistant hnRNPK was overexpressed together with the RNAi targeting the coding sequence a strong depletion of the Myc staining was observed (Fig. 4-IV). After transfection of rat primary hippocampal neurons (DIV21) with the hnRNPK RNAi or the Abi-RNAi constructs (empty pSuper vector as control) or double transfections with the combinations of hnRNPK-RNAi against the coding sequence and the non-resistant or the resistant hnRNPK-Myc constructs, we thoroughly analyzed the morphology of neurons (Fig. 4B-I). First, we evaluated the number of total branching points within the dendritic compartment of transfected neurons. We found a significant increase of branching points in the Abi-1-RNAi, hnRNPK-RNAi as well as in the RNAi plus non-resistant construct groups (Fig. 4B-II). To further investigate this phenotypical alteration, we analyzed the number of different types of dendrites per neuron by counting primary, secondary, tertiary and quartary dendrites. Again, we observed that RNAi mediated knockdown of Abi-1 as well as of hnRNPK significantly shifted the morphology of the dendritic tree towards small, filopodia-like quartary dendrites (Fig. 4B-III).

### Effects of hnRNPK knockdown on synaptogenesis

**Figure 5:**
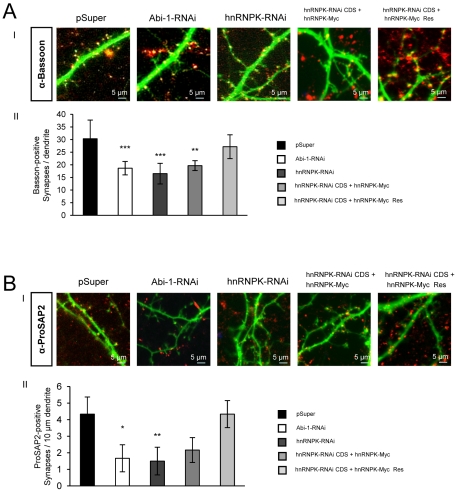
Downregulation of hnRNPK reduces the number of mature synaptic contacts in hippocampal neurons. (AI,II) Decrease in mature synaptic contacts after Abi-1- or hnRNPK-RNAi knockdown. There is a significant decrease in the number of mature synapses with bassoon-positive presynaptic counterparts in the neurons transfected with Abi-1- or hnRNPK-RNAi constructs compared to vector control. At the same time, a more filopodia-like phenotype of the dendritic tree can be observed after RNAi knockdown of Abi-1 or hnRNPK. The RNAi resistant construct is able to rescue the observed reduction. Scale bars are as indicated.(BI,II) Analysis of the reduction of excitatory synapses by using ProSAP2/Shank3 antibodies that label postsynaptic densities (PSDs) reveals a comparable reduction of postsynaptic specializations that is also rescued by the RNAi resistant construct.

Next, we counted the number of mature synapses with bassoon-positive presynaptic or postsynaptic ProSAP2/Shank3 positive counterparts in the transfected neurons, since downregulation of Abi-1 has already been shown to exert an effect on synapse number and maturation. Interestingly, the knock-down of hnRNPK-RNAi mimicked the known phenotype of Abi-1-RNAi by significantly lowering the number of mature synapses of the transfected neurons (Fig. 5A,B). These effects could be rescued by cotransfection of an RNAi resistant hnRNPK construct.

## Discussion

In the study presented, we show that Abi-1 and hnRNPK interact at postsynaptic sites via defined protein-protein interaction motifs and that protein concentrations of both molecules have an impact on neuronal phenotype, especially with respect to the morphology of the dendritic tree and synapse formation. Up to now, hnRNPK has been characterized as a heterogeneous nuclear ribonucleoprotein (hnRNP) particle component being involved in a plethora of fundamental biological processes like transcription modulation, translation, mRNA transport and signal transduction [15], [40]. Despite this functional importance and diversity, hnRNPK expression is cell type specific and depending upon developmental stages. During early stages of mammalian brain development, hnRNPK mRNA is expressed in several brain regions, at later stages, however, the expression seems to be restricted to cortical and hippocampal areas [41]. We could confirm and extend these data by showing that in the adult brain, hnRNPK protein is predominantly found in the cerebellum, the cortex and in the hippocampal formation. Immunohistochemistry as well as Western blot analyses indicate that expression levels are downregulated during postnatal rat brain maturation, supporting the idea that hnRNPK is especially important during neuronal differentiation and early synapse formation. Moreover, we analyzed the subcellular distribution of hnRNPK in neurons. The close analysis of primary hippocampal neurons in culture revealed that hnRNPK is predominantly localized in the nuclear compartment. We were further able to show for the first time that is also distributed within the cytoplasm and can be specifically labeled within the postsynaptic density (PSD).

Here it interacts with Abi-1 and the binding is mediated by the *src-homology 3* (SH3) domain of Abi-1 and the hnRNPK-KI (K interaction) domain. The KI domain of hnRNPK displays at least 3 proline rich clusters that most likely mediate the specific interaction with the Abi-1 SH3 domain as been shown for several other SH3 interaction partners [42]. In hippocampal cells, we could further show by a series of transfection experiments that this interaction is sufficient to localize a truncated hnRNPK fusion protein solely encoding the hnRNPK-KI domain, to Abi-1 positive synaptic sites. Interestingly, this domain is also responsible for several other protein-protein interactions and includes defined phosphorylation sites for ERK and PKC [15], [40]. A previously described interaction between N-WASP and hnRNPK is also mediated via the KI domain and leads to a decrease in N-WASP-mediated filopodia formation [20].

The question arises whether hnRNPK-Abi-1 interaction only takes place at postsynaptic sites. Abi-1 can shuttle between the peripheric and nuclear compartments, depending upon synaptic stimulation [10], [12] and immunocytochemical stainings of hippocampal neurons in this study show both molecules with overlaying fluorescence in dendrites. Transfection experiments in Cos7 cells, however, clearly indicate that hnRNPK is not able to recruit significant amounts of Abi-1 into the nucleus despite the expression of its KNS nuclear shuttling domain. Since in previous experiments, evidence was provided that phosphorylation of Abi-1 by the Abelson kinase is a prerequisite for nuclear entry in neurons [10], it is still conceivable that the described interaction of Abi-1 and hnRNPK also occurs in the nucleus and might be depending upon synaptic stimulation. Furthermore, in cell lines as well as in hippocampal neurons, we found that the knockdown of hnRNPK via RNAi-transfection over 3 days leads to a significant reduction of synaptic contacts in hippocampal neurons that went along with an induction of filopodia-like structures and a more complex dendritic tree. This phenotype is therefore similar to the direct depletion of Abi-1 by RNAi [10] and supports previous data suggesting the role of hnRNPK as an inhibitor of filopodial outgrowth via N-WASP [20]. In neurons, the establishment of a branched actin cytoskeleton is a prerequisite for the development of mature, mushroom-shaped synapses [10]. As neurites during early stages of development, synapses have to self-reshape and mature constantly and exhibit the ability to re-obtain a primitive, filopodia-like state. These reorganizations do take place constantly during the fetal and neonatal period and even adulthood [43], [44]. Abi-1 is an important regulator of filopodial spreading and synaptic maturation by interacting with initiators of the actin polymerization complex, such as N-WASP [13]. In *Xenopus laevis*, hnRNPK depletion prevented the development of axonal processes during early phases of neuronal development [45], [46], [47], and hnRNPK regulates filopodia formation via N-WASP interaction. The association of hnRNPK with the Abi-1 protein might play an important role for those regulatory processes and one could hypothesize a multiprotein complex involving Abi-1, N-WASP and hnRNPK at sites of synaptic maturation with the abiliy to react immediately to changes in the neuronal microenvironment. In this model, hnRNPK might act as a platform molecule for multiple intracellular signaling cascades, leading to its phosphorylation and thus exerting regulatory effects on cytoskeletal reorganization. Recent findings support this hypothesis, since Liebau et al. just showed the importance of such a multiprotein complex including Abi-1 and N-WASP linked to a subgroup of calcium-activated potassium channels, also involved in neural stem cell cytoskeleton rearrangement, for early neurogenesis of rat hippocampal neurons [36], [48].

Here we provide evidence that the postsynaptic interplay between hnRNPK and Abi-1 proteins can effectively influence neuronal morphology including the dendritic structure as well as the synaptic shape. Since Abi-1 is linked to the actin cytoskeleton in spines and PSDs, our findings suggest a multiprotein regulatory complex that enables a fine-tuned control of synaptic maturation and plasticity.

## References

[1] Scheiffele P (2003). Cell-cell signaling during synapse formation in the CNS.. Annual review of neuroscience.

[2] Waites CL, Craig AM, Garner CC (2005). Mechanisms of vertebrate synaptogenesis.. Annual review of neuroscience.

[3] Yuste R, Bonhoeffer T (2001). Morphological changes in dendritic spines associated with long-term synaptic plasticity.. Annual review of neuroscience.

[4] Yuste R, Bonhoeffer T (2004). Genesis of dendritic spines: insights from ultrastructural and imaging studies.. Nature reviews Neuroscience.

[5] Hering H, Sheng M (2001). Dendritic spines: structure, dynamics and regulation.. Nature reviews Neuroscience.

[6] Hotulainen P, Hoogenraad CC (2010). Actin in dendritic spines: connecting dynamics to function.. The Journal of cell biology.

[7] Kiraly DD, Eipper-Mains JE, Mains RE, Eipper BA (2010). Synaptic plasticity, a symphony in GEF.. ACS chemical neuroscience.

[8] Matus A (2000). Actin-based plasticity in dendritic spines.. Science.

[9] Biesova Z, Piccoli C, Wong WT (1997). Isolation and characterization of e3B1, an eps8 binding protein that regulates cell growth.. Oncogene.

[10] Proepper C, Johannsen S, Liebau S, Dahl J, Vaida B (2007). Abelson interacting protein 1 (Abi-1) is essential for dendrite morphogenesis and synapse formation.. The EMBO journal.

[11] Courtney KD, Grove M, Vandongen H, Vandongen A, LaMantia AS (2000). Localization and phosphorylation of Abl-interactor proteins, Abi-1 and Abi-2, in the developing nervous system.. Molecular and cellular neurosciences.

[12] Echarri A, Lai MJ, Robinson MR, Pendergast AM (2004). Abl interactor 1 (Abi-1) wave-binding and SNARE domains regulate its nucleocytoplasmic shuttling, lamellipodium localization, and wave-1 levels.. Molecular and cellular biology.

[13] Innocenti M, Gerboth S, Rottner K, Lai FP, Hertzog M (2005). Abi1 regulates the activity of N-WASP and WAVE in distinct actin-based processes.. Nature cell biology.

[14] Le Clainche C, Carlier MF (2008). Regulation of actin assembly associated with protrusion and adhesion in cell migration.. Physiological reviews.

[15] Bomsztyk K, Denisenko O, Ostrowski J (2004). hnRNP K: one protein multiple processes.. BioEssays : news and reviews in molecular, cellular and developmental biology.

[16] Fenn S, Du Z, Lee JK, Tjhen R, Stroud RM (2007). Crystal structure of the third KH domain of human poly(C)-binding protein-2 in complex with a C-rich strand of human telomeric DNA at 1.6 A resolution.. Nucleic acids research.

[17] Grishin NV (2001). KH domain: one motif, two folds.. Nucleic acids research.

[18] Michael WM, Eder PS, Dreyfuss G (1997). The K nuclear shuttling domain: a novel signal for nuclear import and nuclear export in the hnRNP K protein.. The EMBO journal.

[19] Mikula M, Dzwonek A, Karczmarski J, Rubel T, Dadlez M (2006). Landscape of the hnRNP K protein-protein interactome.. Proteomics.

[20] Yoo Y, Wu X, Egile C, Li R, Guan JL (2006). Interaction of N-WASP with hnRNPK and its role in filopodia formation and cell spreading.. The Journal of biological chemistry.

[21] Charroux B, Angelats C, Fasano L, Kerridge S, Vola C (1999). The levels of the bancal product, a Drosophila homologue of vertebrate hnRNP K protein, affect cell proliferation and apoptosis in imaginal disc cells.. Molecular and cellular biology.

[22] Messias AC, Harnisch C, Ostareck-Lederer A, Sattler M, Ostareck DH (2006). The DICE-binding activity of KH domain 3 of hnRNP K is affected by c-Src-mediated tyrosine phosphorylation.. Journal of molecular biology.

[23] Habelhah H, Shah K, Huang L, Ostareck-Lederer A, Burlingame AL (2001). ERK phosphorylation drives cytoplasmic accumulation of hnRNP-K and inhibition of mRNA translation.. Nature cell biology.

[24] Lee PT, Liao PC, Chang WC, Tseng JT (2007). Epidermal growth factor increases the interaction between nucleolin and heterogeneous nuclear ribonucleoprotein K/poly(C) binding protein 1 complex to regulate the gastrin mRNA turnover.. Molecular biology of the cell.

[25] Notari M, Neviani P, Santhanam R, Blaser BW, Chang JS (2006). A MAPK/HNRPK pathway controls BCR/ABL oncogenic potential by regulating MYC mRNA translation.. Blood.

[26] Ostareck DH, Ostareck-Lederer A, Wilm M, Thiele BJ, Mann M (1997). mRNA silencing in erythroid differentiation: hnRNP K and hnRNP E1 regulate 15-lipoxygenase translation from the 3′ end.. Cell.

[27] Ostareck-Lederer A, Ostareck DH (2004). Control of mRNA translation and stability in haematopoietic cells: the function of hnRNPs K and E1/E2.. Biology of the cell/under the auspices of the European Cell Biology Organization.

[28] Ostareck-Lederer A, Ostareck DH, Hentze MW (1998). Cytoplasmic regulatory functions of the KH-domain proteins hnRNPs K and E1/E2.. Trends in biochemical sciences.

[29] Ostareck-Lederer A, Ostareck DH, Cans C, Neubauer G, Bomsztyk K (2002). c-Src-mediated phosphorylation of hnRNP K drives translational activation of specifically silenced mRNAs.. Molecular and cellular biology.

[30] Feliers D, Lee MJ, Ghosh-Choudhury G, Bomsztyk K, Kasinath BS (2007). Heterogeneous nuclear ribonucleoprotein K contributes to angiotensin II stimulation of vascular endothelial growth factor mRNA translation.. American journal of physiology Renal physiology.

[31] Thyagarajan A, Szaro BG (2004). Phylogenetically conserved binding of specific K homology domain proteins to the 3′-untranslated region of the vertebrate middle neurofilament mRNA.. The Journal of biological chemistry.

[32] Thyagarajan A, Szaro BG (2008). Dynamic endogenous association of neurofilament mRNAs with K-homology domain ribonucleoproteins in developing cerebral cortex.. Brain research.

[33] Boeckers TM, Liedtke T, Spilker C, Dresbach T, Bockmann J (2005). C-terminal synaptic targeting elements for postsynaptic density proteins ProSAP1/Shank2 and ProSAP2/Shank3.. Journal of neurochemistry.

[34] Carlin RK, Grab DJ, Cohen RS, Siekevitz P (1980). Isolation and characterization of postsynaptic densities from various brain regions: enrichment of different types of postsynaptic densities.. The Journal of cell biology.

[35] Liebau S, Proepper C, Schmidt T, Schoen M, Bockmann J (2009). ProSAPiP2, a novel postsynaptic density protein that interacts with ProSAP2/Shank3.. Biochemical and biophysical research communications.

[36] Liebau S, Steinestel J, Linta L, Kleger A, Storch A (2011). An SK3 Channel/nWASP/Abi-1 Complex Is Involved in Early Neurogenesis.. PloS one.

[37] Schmeisser MJ, Grabrucker AM, Bockmann J, Boeckers TM (2009). Synaptic cross-talk between N-methyl-D-aspartate receptors and LAPSER1-beta-catenin at excitatory synapses.. The Journal of biological chemistry.

[38] Kleger A, Seufferlein T, Malan D, Tischendorf M, Storch A (2010). Modulation of calcium-activated potassium channels induces cardiogenesis of pluripotent stem cells and enrichment of pacemaker-like cells.. Circulation.

[39] Liebau S, Propper C, Bockers T, Lehmann-Horn F, Storch A (2006). Selective blockage of Kv1.3 and Kv3.1 channels increases neural progenitor cell proliferation.. Journal of neurochemistry.

[40] Bomsztyk K, Van Seuningen I, Suzuki H, Denisenko O, Ostrowski J (1997). Diverse molecular interactions of the hnRNP K protein.. FEBS letters.

[41] Blanchette AR, Fuentes Medel YF, Gardner PD (2006). Cell-type-specific and developmental regulation of heterogeneous nuclear ribonucleoprotein K mRNA in the rat nervous system.. Gene expression patterns : GEP.

[42] Feng S, Chen JK, Yu H, Simon JA, Schreiber SL (1994). Two binding orientations for peptides to the Src SH3 domain: development of a general model for SH3-ligand interactions.. Science.

[43] Hering H, Sheng M (2003). Activity-dependent redistribution and essential role of cortactin in dendritic spine morphogenesis.. The Journal of neuroscience : the official journal of the Society for Neuroscience.

[44] Liebau S, Vaida B, Storch A, Boeckers TM (2007). Maturation of synaptic contacts in differentiating neural stem cells.. Stem cells.

[45] Iwasaki T, Koretomo Y, Fukuda T, Paronetto MP, Sette C (2008). Expression, phosphorylation, and mRNA-binding of heterogeneous nuclear ribonucleoprotein K in Xenopus oocytes, eggs, and early embryos.. Development, growth & differentiation.

[46] Liu Y, Gervasi C, Szaro BG (2008). A crucial role for hnRNP K in axon development in Xenopus laevis.. Development.

[47] Marcu A, Bassit B, Perez R, Pinol-Roma S (2001). Heterogeneous nuclear ribonucleoprotein complexes from Xenopus laevis oocytes and somatic cells.. The International journal of developmental biology.

[48] Liebau S, Vaida B, Proepper C, Grissmer S, Storch A (2007). Formation of cellular projections in neural progenitor cells depends on SK3 channel activity.. Journal of neurochemistry.

